# The relationship between computed tomography appearance of pulmonary tuberculosis and blood glucose levels in 763 diabetes mellitus patients with pulmonary tuberculosis: a comparative study

**DOI:** 10.1007/s12020-022-03033-8

**Published:** 2022-03-24

**Authors:** Yan Ren, Hongmei Ren, Qian Tian, Xue Li, Yuxi Liu

**Affiliations:** Tuberculosis Ward 9, Shenyang Tenth People’s Hospital, Shenyang, 110044 China

**Keywords:** CT appearance, Pulmonary tuberculosis, Blood glucose, Diabetes, Cavities

## Abstract

**Introduction:**

Glucose tolerance is often impaired in pulmonary tuberculosis (TB) patients. We aimed to explore the relationship between computed tomography (CT) findings of TB and blood glucose levels in diabetes mellitus (DM) patients.

**Methods:**

763 diabetes mellitus patients with pulmonary tuberculosis (DMTB) from March 2015 to March 2018 were selected and their clinical data were retrospectively evaluated. CT appearance of DMTB was reviewed and compared according to blood glucose levels and CT scores. TB scores were calculated according to the combination of typical and atypical CT appearance. The relationship between blood glucose levels and CT scores was analyzed via Pearson correlation coefficient.

**Results:**

TB lesions mainly occurred in the left lung and the lower lobes in the DMTB patients. Nodules and cavities are the main typical complications in these DMTB patients, and especially the number and size of cavities predominantly contribute to CT scan scores. The size of cavities (diameters (cm), median (95% CI of median)) was 0.72 (0.66–0.77), 1.20 (1.09–1.28), and 3.45 (2.92–3.94) from the low-, middle- and high-CT-score groups, respectively. The patients with high CT scores had a higher level of fasting plasma glucose (mean 13.48 mM, 95% CI of median 12.56–14.44 mM) than the patients in the low-CT-score (mean 8.73 mM, 95% CI of median 8.49–9.36 mM) and middle-CT-score groups (mean 10.16 mM, 95% CI of median 9.89–10.49 mM) (*P* < 0.0001). CT scores have a consistent relationship with the levels of blood glucose (rho = 0.60, *P* < 0.0001).

**Conclusions:**

CT appearance stands for the severity of tuberculosis and is closely associated with blood glucose levels in diabetic TB patients.

## Introduction

Glucose tolerance may be impaired when the lung injury occurs, such as pulmonary tuberculosis (TB) [1, 2], and is closely associated with the risk of type 2 diabetes mellitus (type-II DM) [3]. On the other hand, diabetic disease will further increase the severity of pulmonary TB if the impaired glucose tolerance impairments cannot be controlled [4]. Type-II DM is regarded as an important factor in the development and progression of pulmonary TB [2, 5]. Radiological presentation (computed tomography, CT) revealed the increase in the number and size of cavities in the lower lung part in TB patients with DM than the patients without DM [3, 6, 7]. Cavitary lesion and more infected lower lobes were also found in TB patients with DM [8]. However, the relationship between the severity of TB and the severity of type-II DM remains widely unclear.

TB severity is linked with the more significant number of cavities and wider cavity lesion extension [9], and the size of cavities [10]. Raised blood glucose levels are a principal risk factor for predicting the severity of DM [11]. Therefore, the number and size of cavities of TB patients with type-II DM may be affected by the blood glucose levels. The aim of the present work was to explore the relationship between CT appearance of pulmonary TB and blood glucose levels in 763 DMTB patients in Shenyang, China.

## Methods

### Participants

Clinical records were from all the patients who visited our hospital from March 2015 to March 2018 with the diagnosis of type-II diabetes mellitus patients with pulmonary tuberculosis (DMTB) were retrospectively evaluated.

### DMTB diagnosis

DMTB was diagnosed according to the Chinese Pulmonary Diagnosis Criteria WS288-2017 [12] and Health Industry Standard of the People’s Republic of China WS196-2017 [13]. The patients had two fasting blood glucose (2FBG) levels of ≥7.0 mM [14]. Mycobacterium tuberculosis (MTB) strain and positive acid-fast bacilli were detected in sputum culture.

### TB score evaluation

TB scores were evaluated according to the previous report, including typical CT appearance (the predominant patchy areas of centrilobular micronodules, the number, and size of nodules, the predominant patchy areas of tree-in-bud lesions, the number and size of cavities, the presence or absence of endobronchial lesion, the number of small airway wall thickening (lumen less than 80% of total), the predominant patchy areas of bronchiolectasis/bronchiectasis) and atypical CT appearance (the predominant patchy areas of interlobular septal thickening, the predominant patchy areas of ground-glass opacities, the size of consolidation, presence or absence of pleural effusion, presence or absence of random micronodules, the predominant patchy areas of clustered micronodules (CMs), presence or absence of reversed halo sign) with the highest scores 45 [15].

### Inclusion criteria

The following patients were included in the present study. The bacterial pathogen Mycobacterium was found in their sputum culture and identified as the strain MTB. The patients were 70> age >40 years diagnosed and on treatment for TB. The patients were diagnosed with type II DM and had 2FBG levels of ≥7.0 mM [14]. All patients had drug susceptibility testing (DST) records. All TB cases were with resistance to Rifampicin (RRTB). All patients had at least visible nodules on CT scans. The DST and strain identification of all patients came from Shenyang city. The patients had 2FBG levels of ≥7.0 mM.

### Exclusion criteria

The following patients were excluded from the present study, including (1) missing CT images; (2) surgical resection of the unilateral lung, and (3) HIV infection.

### Sample size

The sample size was calculated according to a previous report [16]. The percentage of diabetic patients with glycemic control was near 50%. Odds ratio of importance regarding manifestations and outcome of TB was more than 2.5. A 95% 2-sided confidence interval and 80% power were applied, and the acquired number of subjects was more than 336. We found that the number (763) of DMTB patients collected from 2015–2018 was much higher than 336 and planned to consider all DMTB persons because of the uncertainty of assumptions.

### Patients grouping

**Fig. 1:**
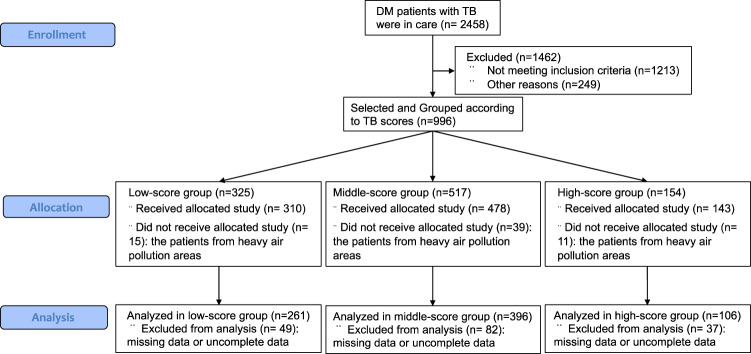
Flowchart of the present retrospective cohort study. Flowchart shows the screening and inclusion process for the patients in this study

We collected the clinical data from 2458 DMTB patients were in care from March 2015 to March 2018. After inclusion and exclusion criteria selection, and other reasons, 1462 patients were excluded. All the patients were assigned into three groups (low, middle, and high-score) based on TB scores. The patients were from heavy air pollution areas (characterized over PM2.5) and with missing data were further removed from the present study. Finally, 261, 396, and 106 patients from low-, middle- and high-score groups, respectively, were selected for further study (Fig. 1).

### Data collection

All posteroanterior and lateral chest roentgenograms were evaluated by two pneumologists in the present work (YR and HR). Tuberculous lesions and cavities were classified as located in the upper or lower lung field when they appeared mostly in the upper or lower half, respectively. A cavitary lesion was considered to be present when its diameter was larger than 2 cm. The difficult CT appearance with doubtful cavities and any discrepancies were cooperatively resolved by the third expert (QT). The data were collected and further analyzed by corresponding experts, including 8 TB experts and 8 DM experts. A final decision was reached via the panel of these experts.

### Statistical analysis

Continuous data in the text and tables were expressed as mean values ± standard deviations (S.D.). Analysis of variance (ANOVA) was used to compare continuous variables among three groups. χ2 test was used for counted number (proportions) comparison among different groups. Pearson correlation coefficient was used to determine the relationship between TB scores and blood glucose levels, and between DM duration and CT scores. There will be a consistent relationship between the two variables if the rank nonparametric correlation (Rho) is more than 0.6 [17]. Statistical tests were performed using SPSS v20.0 (SPSS Inc., Chicago, IL, USA). Statistical significance was set at two-tailed *P* < 0.05.

## Results

### Baseline characteristics

After the selection of including and excluding criteria, a total of 763 DMTB patients were included in the study (Table 1). CT scores were found to be positively associated with serum levels of cholesterol, TG, and LDL and their levels reached the highest level in the high-CT-score group (Table 1, *P* < 0.0001). CT scores were also found to be positively associated with the levels of leukocytes and lymphocytes, and their levels reached the highest level in the high-CT-score group (Table 1, *P* < 0.0001). CT scores were affected by TB and type-II DM duration (Table 1, *P* < 0.0001). Furthermore, the uncontrolled/controlled type-II DM cases (ratio) were 65/196 (0.33), 189/207 (0.91) and 69/37 (1.86) (Table 1, *P* < 0.0001) from low-, middle- and high-score groups, respectively. The results suggest that high CT scores are closely associated with the ratio of uncontrolled/controlled type-II DM cases.

**Table 1:** Baseline characteristics among three groups

Parameters	Low-score group (*n* = 261)	Middle-score group (*n* = 396)	High-score group (*n* = 106)	*P* values
Age (years), median (95% CI of median)	54.7 (52.4–56.0)	53.6 (51.0–53.9)	54.9 (51.0–58.0)	0.228
Male/female	226/45	343/53	89/17	0.485
Smoking, cases (100%)	76 (29.12)	118 (29.8)	32 (30.19)	0.973
Chronic renal failure, cases (100%)	27 (10.34)	42 (10.61)	12 (11.32)	0.963
Previous history of malignancy, cases (100%)	16 (6.13)	28 (7.07)	9 (8.49)	0.716
Connective tissue disease, cases (100%)	12 (4.6)	17 (4.29)	8 (7.55)	0.370
Previous history of organ transplantation, cases (100%)	4 (1.53)	7 (1.77)	2 (1.89)	0.963
Cholesterol (mg/dL), median (95% CI of median)	193.7 (191.0–197.2)	200.0 (197.4–202.2)	216.3 (210.8–221.9)	<0.0001
TG (mg/dL), median (95% CI of median)	209.6 (206.9–211.2)	229.7 (225.1–231.7)	237.1 (229.9–244.4)	<0.0001
HDL (mg/dL), median (95% CI of median)	53.4 (52.5–54.8)	53.5 (52.4–54.2)	53.9 (52.5–56.4)	0.736
LDL (mg/dL), median (95% CI of median)	154.4 (151.5–156.6)	165.1 (161.9–166.7)	174.3 (168.9–181.6)	<0.0001
Duration of tuberculosis (years)	1.88 (1.80–1.92)	2.19 (2.13–2.24)	2.97 (2.90–3.09)	<0.0001
Duration of type-II DM (years), median (95% CI of median)	9.85 (9.38–10.30)	10.27 (9.89–10.65)	10.87 (10.21–11.54)	<0.0001
Uncontrolled/controlled type-II DM (ratio)	65/196 (0.33)	189/207 (0.91)	69/37 (1.86)	<0.0001
Leukocytes (cells/mm3)	10,202 ± 634	11,822 ± 1042	14,752 ± 1299	<0.0001
Lymphocytes (cells/mm3)	2118 ± 68	2297 ± 108	2495 ± 141	<0.0001

Note: Low-score group (*n* = 261, CT scores (5–8)), Middle-score group (*n* = 396, CT scores (9–12)), and High-score group (*n* = 106, CT scores (13–16)). *TG* Total glycerol; *LDL-C* Low-density lipoprotein cholesterol, and *HDL-C* High-density lipoprotein cholesterol. There are significant differences if *P* < 0.05

The statistical differences were insignificant for other parameters, including gender distribution, smoking, the cases of chronic renal failure, previous history of malignancy, connective tissue disease, previous history of organ transplantation and serum HDL levels (Table 1, *P* > 0.05).

Among all the patients, solitary pulmonary nodules (SPNs) were the most prevalent diagnostic CT abnormality but the size of most SPNs was less than 1 cm and their numbers were more than 5. The number and size of cavities in the high-CT-score group were more than those in the middle-CT-score and low-CT-score group (Table 2, *P* < 0.0001), and the diameter size of most cavities was less than 3 cm. The size of cavities (diameters (cm), median (95% CI of median)) was 0.72 (0.66–0.77), 1.20 (1.09–1.28), and 3.45 (2.92–3.94) from the low-, middle- and high-CT-score groups, respectively (Table 2). The incidence of bronchiectasis, TB consolidation, and cluster micronodules in the high-CT-score group were more than those in the middle-CT-score and low-CT-score group (Table 2, *P* < 0.0001). The statistical differences were insignificant among the three groups for the incidence of tree-in-bud lesions, centrilobular micronodules, pleural effusion, small airway wall thickening, ground-glass opacities and random micronodules (Table 2, *P* > 0.05). Nodules (> 1 cm), interlobular septal thickening and reversed halo signs were not found in all patients (Table 2).

**Table 2:** CT findings among three groups

(1) Typical CT findings	Low-score group (*n* = 261)	Middle-score group (*n* = 396)	High-score group (*n* = 106)	*P* values
Centrilobular micronodules (<3 mm), cases (%)	20 (7.66)	18 (4.55)	4 (3.77)	0.161
Nodules (3–10 mm), cases (100%)	261 (100)	396 (100)	106 (100)	
Nodules (>1 cm)				
Tree-in-bud lesions, cases (%)	59 (22.6)	73 (18.4)	26 (24.5)	0.251
Cavities, number (%)	133 (51)	424 (107.1)	159 (150)	<0.0001
Cavities, diameters (cm), median (95% CI of median)	0.72 (0.66–0.77)	1.20 (1.09–1.28)	3.45 (2.92–3.94)	<0.0001
Endobronchial lesion				
Small airway wall thickening (lumen <80% of total), cases (%)	16 (6.1)	29 (7.3)	7 (6.6)	0.241
Bronchiolectasis/Bronchiectasis, cases (%)	11 (4.2)	15 (3.8)	25 (23.6)	<0.0001
(2) Atypical CT findings				
Interlobular septal thickening				
Ground glass opacities, cases (%)	5 (1.9)	10 (2.5)	3 (2.8)	0.830
Consolidation, cases (%)	31 (11.9)	49 (12.4)	37 (34.9)	<0.0001
Pleural effusion, cases (%)	13 (5)	15 (3.8)	6 (5.7)	0.623
Random micronodules (<3 mm), cases (%)	16 (6.1)	19 (4.8)	8 (7.5)	0.504
Cluster micronodules, cases (%)	28 (10.7)	47 (11.9)	29 (27.4)	<0.0001
Reversed halo sign				

Data in parentheses are percentage indicated otherwise. *IB* Infectious bronchitis, *TB* Tuberculosis, *PCR P*olymerase chain reaction. *Values are presented as arithmetic mean ± standard deviation (S.D.)

### Characteristics of CT findings

**Fig. 2:**
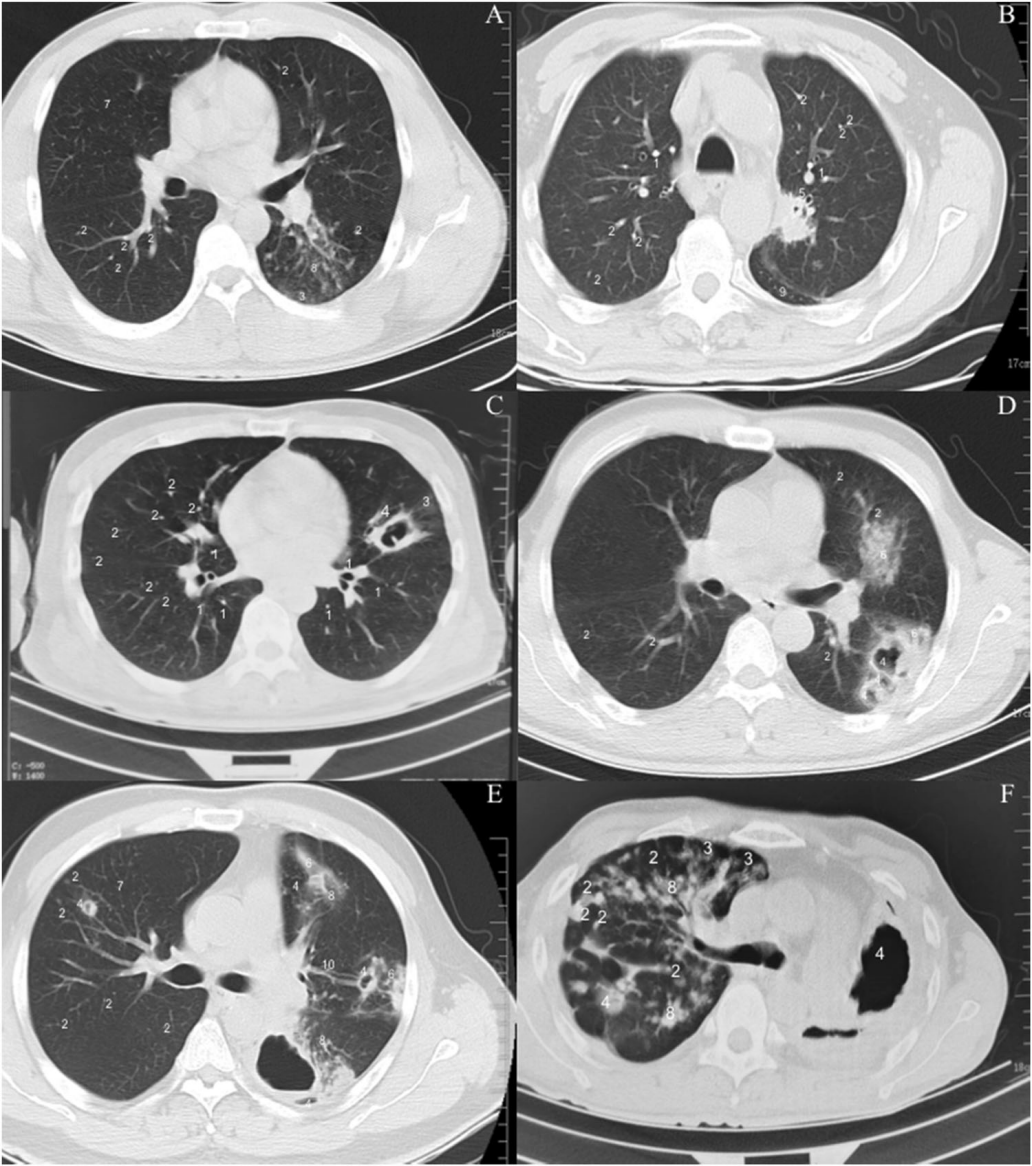
Examples of different Computed Tomography (CT) patterns in pulmonary tuberculosis patients with diabetes mellitus. **A** a 29-year-old male had a cough and sputum for 2 months, which were worsened with fever for 3 d. The patient had developed cough, white sticky sputum, and hemoptysis. The patient was diagnosed with tuberculosis. He had no high blood pressure, no diabetes, and no coronary heart disease. **B** A 58-year-old patient had intermittent cough, sputum, and chest tightness for 3 months. Shortness of breath would occur after activity with uncomforted left chest. Lung CT examination revealed pleural effusion on the left side. She had symptoms of fatigue, poor diet and sleep, and her weight has dropped by about 20 kg in the past 2 months. She had a history of diabetes for 10 years, and took glipizide. She had a history of hypertension for 10 years, and orally administrated with amlodipine mesylate. She had no history of coronary heart disease, no hepatitis, no recognition of surgery no trauma, and no drug and food allergies. **C** A 43-year-old patient had cough, sputum, and fatigue for 5 months, and poor appetite and chest tightness for 3 days. Weight loss was obvious due to high levels of blood glucose levels. The patient had positive purified protein derivative (PPD) test and anti-Mycobacterium tuberculosis IgG antibodies. Pulmonary CT scan showed a big cavity in the left lung. Left and right lungs had some scattered nodules. The patient has been diagnosed with diabetes for 15 years, and has not been treated regularly for blood sugar reduction. He had no high blood pressure, no history of coronary heart disease, no surgery or trauma, no drug or food allergies, and no liver disease. **D** A 53-year-old patient had intermittent cough and sputum for more than 3 months, and worsened for 5 d. Normal exercise would cause shortness of breath and occasional fatigue. The patient was diagnosed with tuberculosis and bronchus tuberculosis and treated with isoniazid, ethambutol, pyrazinamide, and rifampicin. He had the history of diabetes for more than 3 months, and metformin and acarbose were used to control blood glucose level. He had no high blood pressure, or coronary heart disease. **E** A 42-year-old patient had cough, sputum, blood in sputum, and fever for 4 d accompanied by chills and fever. The highest body temperature was 41 °C without regularity. TC showed CMs, nodules and consolidation with unclear boundary in both lungs. Some lesions showed punctate calcification, stretched bronchi and light transmissive areas. The patient was diagnosed with tuberculosis and had the history of 5-year diabetes. At present, insulin was used and blood glucose control was weak. **F** A 39-year-old patient had intermittent cough, sputum expectoration, and shortness of breath for 1 year, and became worse for 1 week. The patient was diagnosed with pulmonary tuberculosis in January 2019, and was treated with rifampicin, pyrazinamide, and levofloxacin for 2 months, and the drug was discontinued by himself. In September 2019, July and October 2020, the disease relapsed, and the anti-tuberculosis treatments included pacifastin, rifampicin, isoniapene ethambutol, oxazinamide, levofloxacin, and amikacin. The whole course of treatment was discontinued by himself. More than 4 months ago, the cough and sputum were aggravated with yellow sputum mainly in the middle night. He had shortness of breath with poor sleep at night and became aggravated after activities. Due to poor compliance of the patient, irregular anti-tuberculosis treatment was performed. He had no history of hepatitis, or high blood pressure, or traumatic surgery, or blood transfusion, and or drug allergy. TB scores were evaluated according the previous report, including typical CT appearance (the predominant patchy areas of centrilobular micronodules, the number and size of nodules, the predominant patchy areas of tree-in-bud lesions, the number and size of cavities, the presence or absence of endobronchial lesion, the number of small airway wall thickening (lumen less than 80% of total), the predominant patchy areas of bronchiolectasis/bronchiectasis) and atypical CT appearance (the predominant patchy areas of interlobular septal thickening, the predominant patchy areas of ground glass opacities, the size of consolidation, presence or absence of pleural effusion, presence or absence of random micronodules, the predominant patchy areas of clustered micronodules (CMs), presence or absence of reversed halo sign) with the highest scores 45 (15). 1, Centrilobular micronodules (<3 mm); 2, Nodules (3–10 mm); 3, Tree-in-bud lesions; 4, Cavity; 5, Small airway wall thickening (lumen < 80% of total); 6, Consolidation; 7, Random micronodules (<3 mm); 8, Cluster micronodules (CMs); 9, Pleural effusion and 10, bronchiectasis

The severity of TB is difficult to be displayed by using a unique criterion. In the present study, the severity of TB was presented by using CT scores via the combination of typical CT findings and atypical CT findings according to the previous report [15]. In the low-CT-score group, TB patients had a lower incidence of upper lung field lesions in comparison with lower lung field lesions (42 cases, 16.1% vs. 153 cases, 58.6%, respectively, *P* < 0.0001). The differences for the distribution ratio were also found in the middle-CT-score group (87 cases, 22.0% vs. 213 cases, 53.8%, respectively, *P* < 0.0001) and the high-CT-score group (23 cases, 21.7% vs. 60 cases, 56.6%, respectively, *P* < 0.0001). With the CT scores increasing, the lesion expanded more lung fields and the upper lung field increased (Fig. 2). In this sense, upper lung field lesions were more likely to exist in patients with middle-CT and high-CT scores. On the other hand, for lower lung field, as well as for upper + lower lung fields, the statistical differences were insignificant in the incidence of unilateral/bilateral lesions among all groups (Fig. 2, *P* = 0.415).

Pulmonary cavities are the definitive mark of TB and the sites with high mycobacterial burden because of drug resistance and treatment failure [18]. However, pulmonary cavities were seldomly found in the low-CT-score group (Fig. 2A, 7 scores and Fig. 2B, 6 scores). Pulmonary cavities were mostly found in middle-CT-score and high-CT-score groups. In some cases, the diameter of the cavity was more than 3 cm and the thickness of the cavity was more than 1 cm (Fig. 2C, 9 scores). Multiple cavities were often found in the middle-CT-score group (Fig. 2D, 12 scores, 3 cavities with the diameters of 2.6 cm, 1.8 cm, and 1.3 cm, respectively. The thickness of cavities is 0.3 cm, 0.5 cm and 0.3 cm, respectively). The larger size of cavities was often found in high-CT-score group (Fig. 2E, 15 scores, 3 cavities; One cavity was located in the right upper lobe with a length of about 1.1 cm and a thickness of 0.5 cm; One cavity was located in the left upper lobe cavity with a length of about 1.7 cm and a thickness of about 0.5 cm; One cavity was located in the left lower lobe with a length of about 4.9 cm and a thickness of about 0.4 cm). In some cases, the size of cavity was more than 7 cm in the high-CT-score group (Fig. 2F, 13 scores, the diameter of cavity was 7.6 cm and its thickness was 0.9 cm). These results suggest that cavity occurrence is the predominant factor for increasing the scores of TB. Multiple cavitary lesions were relatively frequent in the middle-CT-score and high-CT-score groups. CMs exhibit large nodular opacity and are active TB [15], and found in almost half of the patients (364 cases, 47.7%, Fig. 2A, E, F).

**Fig. 3:**
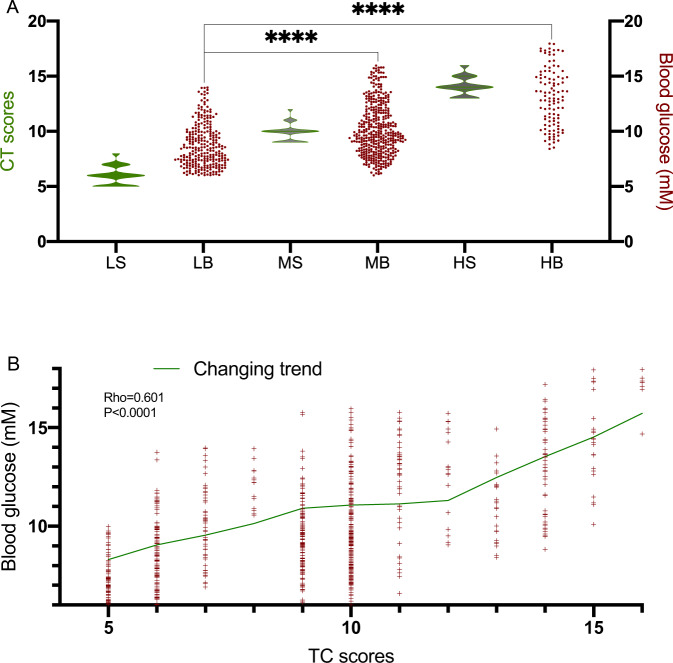
The relationship between blood glucose levels and CT scores. **A** blood glucose levels and CT scores among different groups. LS stands for CT scores in low-CT-score group; LB stands for blood glucose levels in low-CT-score group; MS stands for CT scores in middle-CT-score group; MB stands for blood glucose levels in middle-CT-score group; HS stands for CT scores in high-CT-score group; HB stands for blood glucose levels in high-CT-score group. **B** The relationship between blood glucose levels and CT scores was analyzed based on the Pearson correlation coefficient (PCC). Low-score group (*n* = 261, CT scores (5–8)), Middle-score group (*n* = 396, CT scores (9–12)) and High-score group (*n* = 106, CT scores (13–16)). Spearman rho, rank correlation coefficient. Spearman’s rho > 0.6 shows a consistent relationship between two variables. ****P < 0.0001 vs the low-CT-score group

Blood glucose levels (2FBG) in the high-CT-score TB group (mean 13.48 mM, 95% CI of median 12.56–14.44 mM) were higher than in the middle-CT-score TB group (mean 10.16 mM, 95% CI of median 9.89–10.49 mM), whose blood glucose levels were also higher than the low-CT-score TB group (mean 8.73 mM, 95% CI of median 8.49–9.36 mM) (Fig. 3A, *P* < 0.0001). The Pearson Correlation Coefficient is the methodology normally used for linear variable selection. The analysis showed that blood glucose level was increased with the CT score increasing and had a consistent relationship with the CT scores since the rho value was 0.6 (Fig. 3B, *P* < 0.0001). Pearson correlation coefficient analysis showed that DM duration had a weak but positive correlation with CT scores because the rho value was 0.27 (*P* < 0.0001) [19].

## Discussion

The results are consistent with the previous reports that the severity of TB and DM are mutual affected [20]. Normally, TB is observed predominantly in lung apices while TB tended to occur predominantly in the lower lobes in the DMTB patients (Fig. 2) [21]. However, the exact molecular mechanism for causing the differences remain unclear.

The present findings demonstrated that a low proportion (Fig. 2) of DMTB patients had combined upper and lower lesions of the lungs, which may imply extremity affection caused by MTB complex and seldom reported although the symptom has been reported for more than 100 years [22]. Furthermore, the areas of left pulmonary lesions were bigger than the right ones (Fig. 2).

TB scores were mainly evaluated according to typical CT appearance (the predominant patchy areas of centrilobular micronodules, the number and size of nodules, the predominant patchy areas of tree-in-bud lesions, the number and size of cavities, the presence or absence of endobronchial lesion, the number of small airway wall thickening, the predominant patchy areas of bronchiolectasis/bronchiectasis) and atypical CT appearance (the predominant patchy areas of interlobular septal thickening, the predominant patchy areas of ground-glass opacities, the size of consolidation, presence or absence of pleural effusion, presence or absence of random micronodules, the predominant patchy areas of CMs, presence or absence of reversed halo sign) while cavities are the predominant clinical CT appearance. Therefore, the number and size of pulmonary cavities mainly contribute to the TB scores. It is relatively rare for that disappearance of cavities associated with pulmonary fungal infections [23]. Pulmonary cavities are often considered as pathognomonic caused by pulmonary aspergillosis infection [24]. Cavities make the diagnostic suspicion of TB feasible, and the DM patients with cavitary lesions appearing in one or both lung fields should be regarded the possibility TB [6].

CM often grows over a few months to years, and finally becomes spread bronchogenic lesions. Normally, small CMs are non-caseating granulomas located at the peribronchiolar interstitium, whereas larger CMs are peribronchiolar caseating granulomas, which will invade the airway and lung parenchyma. Although a few patients with CMs were observed in the present work, CMs pretty likely develop from less-invasive non-caseating granulomas to invasive caseating ones [15].

CT scores have a consistent relationship with blood glucose levels (Fig. 3), suggesting that high levels of blood glucose may be a predominant factor for causing manifestations of TB. The increase in blood glucose levels will induce the risk of type-II DM, which have been detected in the patients with multidrug-resistant TB. High levels of blood glucose should be especially controlled to avoid the development of further complications in TB since the increases in glucose levels induce TB susceptibility [25, 26]. DM often results in the poor condition of malnutrition, which will lower the immune status by decreasing the lymphocytes production, IFN-gamma and IL-2 level, and increasing the levels of TGF-beta. Uncontrolled blood glucose has been found to be closely associated with TB incidence [2]. TB is an infectious disease caused by MTB, and high blood glucose in DM patients will increase the possibility of susceptibility to TB bacteria and longer duration therapy [27].

Atypical findings in DMTB patients greatly contribute to the diagnostic CT scan scores in the present study. Conventionally, atypical radiologic manifestations of TB are prevalent in immunocompromised patients, with a propensity to evolution quickly [28]. CM is a main atypical manifestation of TB [29] and should pay more effort and attention without considering whether it is single localization or multiple distributions since it is a more common and atypical form of secondary TB [30].

### The strengths of the present study

Our study included 763 DMTB patients, which occupy the largest sample collected in a single study. This allowed us to draw a certain conclusion to better understand the relationship between the severity of TB and DM among DMTB patients by comparing the TB scores and blood glucose levels. CT scores have a consistent relationship with blood glucose levels, which may be a predominant factor for causing manifestations of TB. High levels of blood glucose should be controlled to avoid the development of further complications in TB.

### The limitations in the present study

Missing data are most common confronted in retrospective studies which will compromise the results of statistical inference and affect the final conclusion in the present study. Although CT has been used as the main tool for the diagnosis and prognosis of TB, it is still difficult to obtain a correct estimation of the clinical symptoms of TB. CT technology is limited with a reduced overall sensitivity since the technology only depends on morphological and dimensional criteria. Photon-counting CT scan provides better spatial and energy resolution, which can show molecular imaging of TB [31]. Furthermore, in recent years, multidetector technology has been implemented for differentiating primary progressive TB [32]. All these new technologies should be considered in the future work. On the other hand, the scoring method has its limitations in assessing subtle changes of TB, especially for the lesions that may not share a similar scoring method. The inclusion may be imperfect because we guess some TB may be caused by air pollution or combination of air pollution and DM, but we cannot be sure of that. These patients from heavy air pollution areas (characterized by over PM2.5) were excluded.

## Conclusion

Blood glucose level has a consistent relationship with the CT scores, suggesting that DM is closely associated with complications of TB in DMTB patients. The patients with high CT scores have a higher level of 2FBG than the patients in the low-CT-score and middle-CT-score groups. The size of cavities (diameters (cm)) is increased from the low-, middle- to high-CT-score groups. Further work needs to confirm whether blood glucose level affects the number and size of TB cavities.

## Data Availability

Some or all datasets generated during and/or analyzed during the current study are not publicly available but are available from the corresponding author on reasonable request.
